# Growth, Structure and Optical Characterization of Rb_3_Ti_3_P_5_O_20_ Single Crystal

**DOI:** 10.3390/ma15155346

**Published:** 2022-08-03

**Authors:** Jianfu Zhao, Pengfei Zhu, Zhenyan Wang, Li Ai, Xiulan Duan, Fapeng Yu

**Affiliations:** State Key Laboratory of Crystal Materials, Institute of Crystal Materials, Shandong University, Jinan 250100, China; 202012909@mail.sdu.edu.cn (J.Z.); joseph.zhu.nt@gmail.com (P.Z.); wangzhenyan@mail.sdu.edu.cn (Z.W.); 201912593@mail.sdu.edu.cn (L.A.); fapengyu@sdu.edu.cn (F.Y.)

**Keywords:** phosphate, single crystal growth, electronic structure, optical properties

## Abstract

Phosphate crystals attract much attention on account of their rich crystal structures and excellent physical and chemical properties. Herein, Rb_3_Ti_3_P_5_O_20_ single crystals were grown by the high temperature solution method using Rb_2_CO_3_ and NH_4_H_2_PO_4_ as the fluxes. This crystal, with non-centrosymmetric Pca2_1_ space group, presents a three-dimensional framework structure composed of [TiO_6_] octahedron, [PO_4_] tetrahedra, and [P_2_O_7_] dimers. The electronic structure was measured via X-ray photoelectron spectroscopy. The measurements found that Rb_3_Ti_3_P_5_O_20_ has stronger Ti–O ionic bonding properties and weaker P–O covalent bonding properties compared to RbTiOPO_4_. Optical measurements indicated that Rb_3_Ti_3_P_5_O_20_ has a 3.54 eV band gap and a wide transmission range (0.33–4.5 μm). Theoretical calculations showed that Rb_3_Ti_3_P_5_O_20_ crystals have a moderate birefringence of 0.079 at 1064 nm. In addition, the relationship of the structure–property was studied using first-principles method. The results demonstrated that TiO_6_ octahedron played a significant role for the optical properties.

## 1. Introduction

Inorganic phosphate materials have received considerable attention due to their rich structures and excellent physicochemical properties, as well as possessing broad applications in the fields of nonlinear optics, ferroelectricity, luminescence, energy technologies, and catalysts [1,2,3,4,5]. For instance, KTiOPO_4_ (KTP) and KH_2_PO_4_ (KDP) crystals have been used for solid-state lasers as important nonlinear optical materials, and olivine-type LiFePO_4_ is an outstanding cathode material for rechargeable Lithium batteries with high capacity [6,7,8]. In the structure of phosphate materials, one P atom is generally coordinated with four O atoms to shape into the [PO_4_] tetrahedra. These [PO_4_] tetrahedra units are also linked into different phosphorus oxygen groups by sharing oxygen atoms such as bisphosphonates, polyphosphate, and (PO_3_)_∞_ chains. These P–O groups can bond with other ionic polyhedrons to build three-dimensional framework structures [2,4,9].

Among the phosphates, rubidium titanyl phosphate (RTP) crystal has significant technological applications in electro-optic shutters, Q-switches, and pulse selectors based on its large dielectric constant, high laser damage threshold, and stable physicochemical properties [10,11]. The three-dimensional crystal structure of RTP is made up of an alternating connection of [TiO_6_] octahedral groups and [PO_4_] tetrahedral groups, and the resulting cavities are occupied by the alkali metal Rb atoms [12,13]. Research indicates that the large-distortion of [TiO_6_] octahedron in the structure plays a major role in the optical properties of titanium-containing phosphates [14]. As well as RTP, RbTiP_2_O_7_ and RbTi_2_(PO_4_)_3_ materials, containing Rb, Ti, P, and O elements, were previously reported because of the potential technological interest [15,16].

As new kinds of phosphates, Me_3_Ti_3_P_5_O_20_ (Me stands for alkaline-earth metals) materials are non-centrosymmetric and contain [TiO_6_] and [PO_4_] functional units in the structure. K_3_Ti_3_P_5_O_20_ crystal was first reported by Nagornyi et al. in 1993 and showed the potential application as a valuable NLO optical material [17]. Rb_3_Ti_3_P_5_O_20_ crystal is the second material reported for Me_3_Ti_3_P_5_O_20_ compounds, after K_3_Ti_3_P_5_O_20_ [18]. However, there are few studies on Rb_3_Ti_3_P_5_O_20_ crystals. The growth of bulk crystals and the property characterization were not carried out. In this work, Rb_3_Ti_3_P_5_O_20_ single crystals were grown by the high temperature solution growth (HTSG) method and measured via powder X-ray diffraction (XRD) and Infrared (IR) spectroscopy. The electronic structure was studied by X-ray photoelectron spectroscopy (XPS). Linear optical properties and nonlinear optical property (frequency-doubled effect) were investigated. With the aim to better explain the structure–property relation, the density functional theory (DFT) method was applied to compute the density of states (DOS), energy band structure, and linear refractive indices (n).

## 2. Materials and Methods

### 2.1. Single Crystal Growth

Rb_3_Ti_3_P_5_O_20_ single crystals were prepared by the HTSG method. The raw materials TiO_2_ (99.9%, TianJin Chemical Reagent Factory, Tianjin, China), NH_4_H_2_PO_4_ (99.5%, Sinopharm Chemical Reagent Co., Ltd., Shanghai, China), and Rb_2_CO_3_ (99.9%, Jiangxi Dongpeng New Materials Co., LTD., Jiangxi, China) were not subjected to any additional treatment. Rb_2_CO_3_, NH_4_H_2_PO_4_, and TiO_2_ were thoroughly mixed and adequately ground in a molar ratio of 9:20:3 and put into a Pt pot [18]. First, the Pt pot with the batch was heated to 700 °C in the furnace and held for 12 h until all the decomposed gases (NH_3_, CO_2_) were gone. Second, the mixture was put into a programmable temperature vertical resistance wire heating furnace and heated to 950 °C for 48 h. A platinum agitator blade was put into the solution for mechanical stirring for at least 36 h in order to increase the solution homogeneity. Third, the temperature of the solution was slowly decreased to 800 °C over two weeks and finally dropped to room temperature for seven days. Transparent colorless crystals were obtained from the excess flux by washing the cake in water.

### 2.2. Characterization

The XRD pattern was recorded by employing a Rigaku SmartLab 9KW X-ray diffractometer (Tokyo, Japan) set with the use of a Copper target X-ray source (λ = 0.15418 nm) in the diffraction angle range (2θ) of 10–60°. The measurement had a scanning step size of 0.02° and a scanning step time of 0.4 s at room temperature. A bulk transparent Rb_3_Ti_3_P_5_O_20_ single crystal (0.12 × 0.1 × 0.1 mm^3^) was chosen for the analysis of single crystal structure. The single crystal diffraction data collection was recorded via a Bruker D8 VENTURE PHOTON 100 diffractometer (Karlsruhe, Germany) with the use of a monochromatic Molybdenum target X-ray source (λ = 0.71073 Å) at 296.15 K. The crystal structure was resolved with the SHELXT structure solution program via Intrinsic Phasing and polished up with the SHELXL refinement package by making use of least square minimization [19]. The precise crystallographic data and detailed experimental conditions of Rb_3_Ti_3_P_5_O_20_ crystal are listed in Table 1. Electron Probe Microanalysis (EPMA) was measured by a Shimadzu EPMA-1720H (Kyoto, Japan) for compositional analysis of the Rb_3_Ti_3_P_5_O_20_ crystal. A well-polished wafer was chose for measurement. XPS spectra were surveyed by an X-ray photoelectron spectrometer (Thermo Fisher ESCALAB 250, Waltham, MA, USA) in an ultra-high vacuum (<10^−7^ Pa) with the use of a monochromatic aluminum target X-ray source. The excess charging on the sample surface during the measurement was neutralized by the neutralization gun [20].

IR spectra were measured with a Nicolet NEXUS 670 infrared spectrometer (Thermo Nicolet Corporation, Madison, WI, USA). Fully ground crystalline powders were used for testing. Ultraviolet-Visible (UV-Vis) diffuse reflectance data for Rb_3_Ti_3_P_5_O_20_ crystals were obtained by using a Hitachi UH4150 spectrophotometer (200–800 nm, Tokyo, Japan). The optical transmission spectra were measured on a Hitachi UH4150 UV-Vis-IR spectrometer (200–2000 nm) and a Nicolet NEXUS 670 FTIR spectrometer (2000–5000 nm) using a well-polished crystal wafer of Rb_3_Ti_3_P_5_O_20_. The frequency-doubling effect of Rb_3_Ti_3_P_5_O_20_ crystal was tested on a Q-switched Nd:YAG solid-state laser under a 1064 nm wavelength by the Kurtz–Perry technique, and a KDP was used as a reference [21]. The test conditions were not changed after the test start to ensure the accuracy of the results.

### 2.3. Computational Details

The electronic structure of Rb_3_Ti_3_P_5_O_20_ was calculated by the ultra-soft pseudopotential method using DFT in the CASTEP module including density of states (DOS), band structure (Eg) and refractive indices (n) [22]. The 3D crystallographic structure file from single crystal structure determination of Rb_3_Ti_3_P_5_O_20_ crystals was used for optimization and calculation. During the numerical calculation, generalized gradient approximation and Perdew–Burke–Ernzerhof functions were used to optimize the total energy of the system to a minimum. Thereby, the Rb 4p^6^5s^1^, Ti 3p^6^4s^2^3d^2^, P 3s^2^3p^3,^ and O 2s^2^2p^4^ states were considered as the valence electrons. The K point mesh was set as 2 × 6 × 3 in the Brillouin region and the cut-off limit of kinetic energy was set at 350 eV. The refractive indices and birefringence of the Rb_3_Ti_3_P_5_O_20_ crystal were computed based on the obtained band structure and DOS. 

## 3. Results and Discussion

### 3.1. Crystal Growth

**Figure 1:**
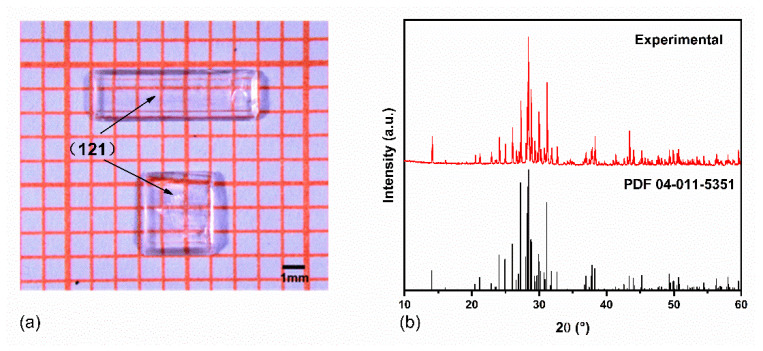
(**a**) The photograph of the Rb_3_Ti_3_P_5_O_20_ single crystals. The crystal faces are all perpendicular to the page (121); (**b**) X-ray powder diffraction pattern of the as-grown Rb_3_Ti_3_P_5_O_20_ single crystal.

**Figure 2:**
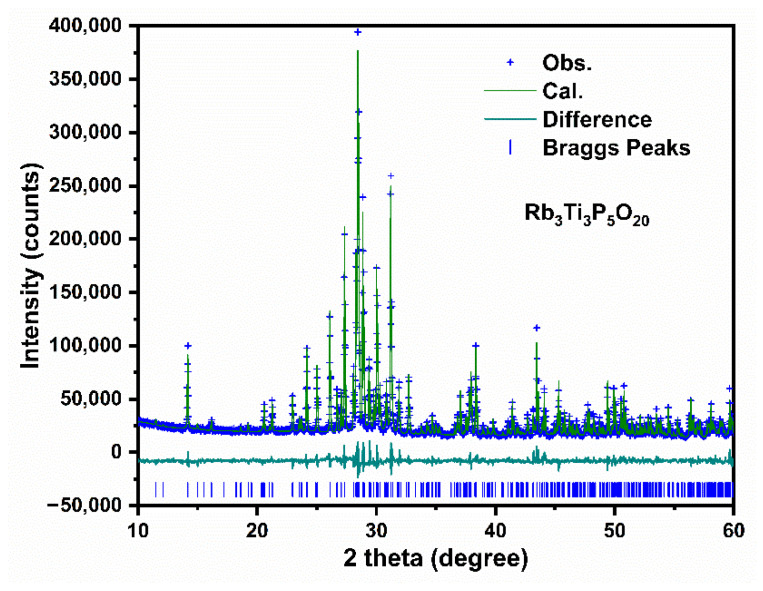
The Rietveld refinement results of Rb_3_Ti_3_P_5_O_20_. Obs. = observed date, Cal. = calculated date, Difference = Obs.−Cal., Braggs Peaks represent the diffractions peak positions.

Due to the high thermal stability of the Rb_3_Ti_3_P_5_O_20_ crystal, it can be grown below the melting point using the high temperature solution method [18]. To obtain high quality crystals, it is extremely vital to choose an appropriate flux. Previously, for decreasing the viscosity of the melt and the melting point, Rb_2_CO_3_ and NH_4_H_2_PO_4_ fluxes were utilized in our laboratory to grow high quality single crystals of pure RTP and doped-RTP [13,23,24]. Therefore, we have chosen Rb_2_CO_3_ and NH_4_H_2_PO_4_ to serve as fluxes for growing Rb_3_Ti_3_P_5_O_20_ single crystals. As a result, the millimeter-sized transparent colorless single crystals were successfully obtained. Figure 1a shows the photograph of as-grown Rb_3_Ti_3_P_5_O_20_ crystals with dimensions of 7 × 2 × 1 mm^3^ and 3 × 3 × 1 mm^3^. The experimental powder XRD of Rb_3_Ti_3_P_5_O_20_ crystal is plotted in Figure 1b. The diffraction peak positions of as-grown crystals correspond well to a standard Rb_3_Ti_3_P_5_O_20_ pattern (PDF No. 82-1169) [18], suggesting that the Rb_3_Ti_3_P_5_O_20_ crystals possess not only high purity but also good crystallinity. Meanwhile, the Rietveld refinement of XRD was carried out by GSAS 2 software and the final plot is shown in Figure 2. The compositional analysis of Rb_3_Ti_3_P_5_O_20_ crystal shows that elemental ratios of Rb, Ti, P, and O are 3.55:3.25:6.12:20.42.

### 3.2. Crystal Structure

**Figure 3:**
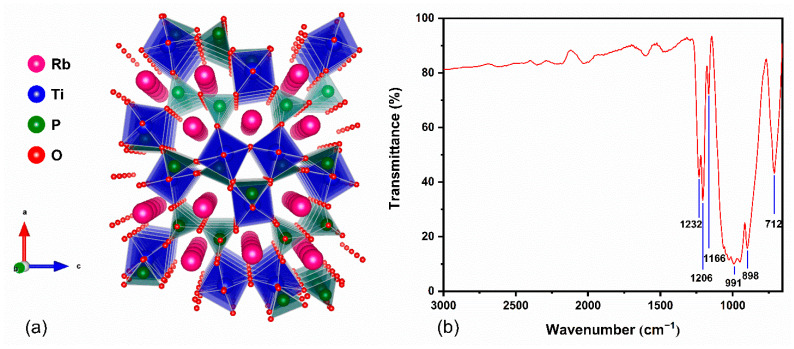
(**a**) The crystal structure of Rb_3_Ti_3_P_5_O_20_ crystal (Rb–O bonds are deleted for clarity); (**b**) IR spectra of Rb_3_Ti_3_P_5_O_20_.

The structure of the Rb_3_Ti_3_P_5_O_20_ crystal was obtained by single crystal XRD analysis. It belongs to an orthogonal crystal system with a polar space group of Pca2_1_ (No. 29) with the unit cell parameters a = 18.2967(17) Å, b = 6.3043(5) Å, c = 14.7942(15) Å, and Z = 4. The unit cell parameters are similar to those reported in a paper (a = 18.282(2) Å, b = 6.2932(7) Å, c = 14.773(2) Å) [18]. The structure diagram of the Rb_3_Ti_3_P_5_O_20_ crystal along the b-axis is plotted in Figure 3a. It features a three-dimensional (3D) structure consisting of [TiO_6_] and [PO_4_] polyhedrons, linked via P–O–P, P–O–Ti and Ti–O–Ti bonds. The Rb atoms are ten-coordinated or eleven-coordinated by oxygen atoms to maintain electrical neutrality, and they are located in the one-dimensional channels. The Ti–O and P–O bond lengths are in the regions 1.797–2.031 Å and 1.483–1.615 Å, respectively. The O–P–O and the O–Ti–O angles in the Rb_3_Ti_3_P_5_O_20_ crystal are in the ranges 98.9°–116.8° and 84.89°–177.95°.

For the purpose of further verifying the types of phosphorus oxygen groups in Rb_3_Ti_3_P_5_O_20_ structure, IR spectra (Figure 3b) were measured for the 600–3000 cm^−1^ wavenumbers. The peaks of 1232, 1206, and 1166 cm^−1^ are attributable to the asymmetric stretching vibrations of P–O bonds. The IR spectra, ranging from 800 to 1100 cm^–1,^ are assigned to the symmetric stretching vibrations and the asymmetric stretching vibrations of P–O–P. The peak of 712 cm^−1^ is caused by the symmetric stretching vibrations of P–O–P. Hence, the IR spectra specify the existence of [PO_4_] tetrahedron and [P_2_O_7_] dimer, which coincide with the results obtained from the single crystal structure analysis of related phosphates [25,26].

### 3.3. Electronic Structure

**Figure 4:**
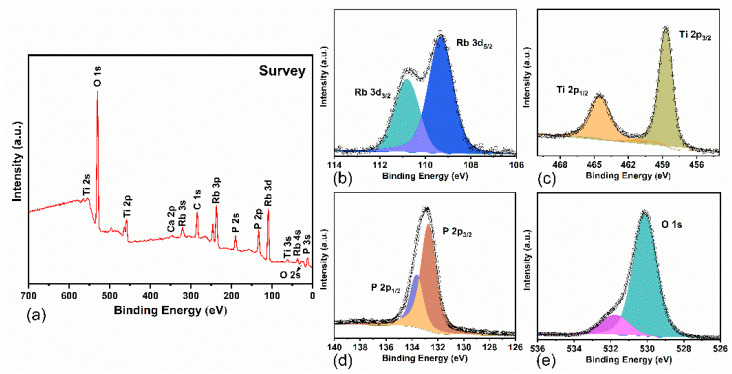
(**a**) Survey XPS spectrum of Rb_3_Ti_3_P_5_O_20_ crystal. The corresponding element peaks are marked in the spectrum; (**b**–**e**) High resolution XPS spectra recorded for Rb_3_Ti_3_P_5_O_20_ crystal.

The electronic structure of the Rb_3_Ti_3_P_5_O_20_ crystal was first measured by XPS and analyzed. The survey spectrum recorded for the Rb_3_Ti_3_P_5_O_20_ single crystal is shown in Figure 4a. The characteristic peaks of all constituent elements (Rubidium, Titanium, Phosphorus, and Oxygen) were found in the survey spectrum. The C 1s peak (284.6 eV) was attributed to hydrocarbonate contamination, and the line was used as a reference for the binding energy scale calibration. The calcium element was also tested as surface contamination.

The deconvoluted high-resolution XPS spectra [27,28], of major elements in Rb_3_Ti_3_P_5_O_20_ crystal are shown in Figure 4b–e. The binding energy (BE) values and the BE difference of the constituent elements of the title compound are presented in Table 2. RbTiOPO_4_ (RTP) and KTiOPO_4_ (KTP) are given for comparison. The BE values of Rb 3d_5/2_, Ti 2p_3/2_, P 2p, and O 1s for Rb_3_Ti_3_P_5_O_20_ crystal are 109.3 eV, 458.6 eV,132.7 eV, and 530.1 eV, respectively. The main peak of O 1s at 530.1 eV corresponds to lattice oxygen. The weak peak at 531.8 eV is usually attributed to defects and contamination (such as H_2_O and CO_2_) of the material surface. The BE values are similar to those of the corresponding elements in RTP and KTP crystals, and this indicates that the atoms are in similar chemical environments [29,30]. The BE difference ΔBE (M–O) = BE (O 1s)—BE(M), where M is the element to be analyzed, is usually used to evaluate the chemical bonding of the elements in crystal lattice because the parameters are insensitive to the surface charging effects [31,32,33]. As for Rb_3_Ti_3_P_5_O_20_, the BE difference values were calculated to be as follows: ΔBE (Ti–O) = 71.5 eV, ΔBE (P–O) = 397.4 eV and ΔBE (Rb–O) = 420.8 eV, respectively. Compared with RTP, Rb_3_Ti_3_P_5_O_20_ shows smaller ΔBE (O–Ti) and ΔBE (O–P) values. The results demonstrate that the Rb_3_Ti_3_P_5_O_20_ crystal exhibits weaker covalency of P–O bonds and stronger ionicity of Ti–O bonds compared with RTP.

### 3.4. Optical Properties Characterization

**Figure 5:**
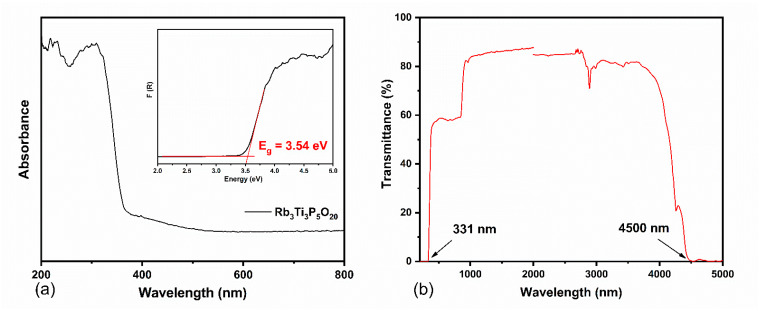
(**a**) UV-Vis diffuse reflectance spectra of Rb_3_Ti_3_P_5_O_20_ crystal. The inset displays the experimental band gap; (**b**) UV-vis and IR transmission spectra of the Rb_3_Ti_3_P_5_O_20_ crystal.

The UV-Vis diffuse reflectance spectrum of Rb_3_Ti_3_P_5_O_20_ crystals is shown in Figure 5a. The absorption data were calculated based on reflection spectra by using the Kubelka–Munk transformation equation:

(1) $$F\left(R\right)=K/S={\left(1-R\right)}^{2}/\left(2R\right)$$

where *S* is the scattering coefficient, *K* is the absorption coefficient, and *R* is the reflectance [34]. The optical band gap of Rb_3_Ti_3_P_5_O_20_ crystals is estimated to be 3.54 eV, which is corresponding to the absorption edge of 339 nm. For further obtaining a precise value of the absorption edge, the transmission spectrum (200–5000 nm) of a well-polished wafer was recorded. Figure 5b demonstrates that the transmission cutoff edge can reach down to 331 nm in the UV region, which is shorter compared with the RTP crystal (350 nm). The IR region cutoff edge of Rb_3_Ti_3_P_5_O_20_ crystal is located at 4.5 μm. This shows the similarity to the KTP and RTP crystals. The Rb_3_Ti_3_P_5_O_20_ crystal is of high transparency in the UV-Vis-NIR region and has a wide optical transparency range of 0.33–4.5 μm. The step near 800 nm is caused by a test instrument (light source change). The absorption band at 3000 nm may be related to the formation of hydrogen bonds in the crystal (stretching vibrations of the O-H bond).

**Figure 6:**
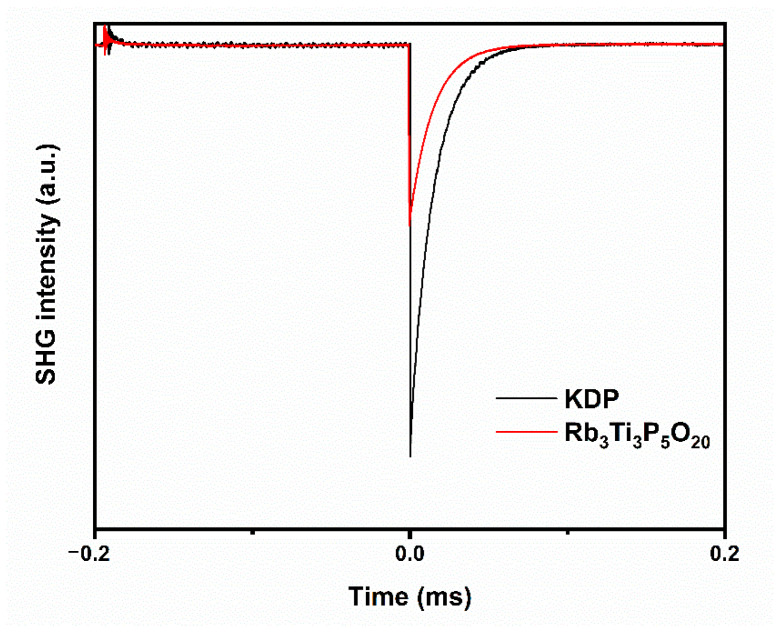
The SHG signals for Rb_3_Ti_3_P_5_O_20_ and KDP standard sample.

The SHG response was measured because Rb_3_Ti_3_P_5_O_20_ crystal is non-centrosymmetric. The result is plotted in Figure 6. The SHG intensity is about 0.4 × KDP for a particle size range of 70–90 μm. The magnitudes of distortions of the TiO_6_ octahedra were calculated using the method proposed by P. Shiv Halasyamani [35]. The magnitude of the distortion was quantified by considering deviations from 180° of the three trans O–Ti–O bond angles as well as the six Ti–O bond distances in the [TiO_6_] octahedra. The calculation formula of the octahedral distortion (Δd) is as follows:

(2) $$\begin{array}{cc} \Delta d= & \frac{\left|\left(M-O1\right)-\left(M-O4\right)\right|}{\left|\cos\theta_{1}\right|}+\frac{\left|\left(M-O2\right)-\left(M-O5\right)\right|}{\left|\cos\theta_{2}\right|}+\frac{\left|\left(M-O3\right)-\left(M-O6\right)\right|}{\left|\cos\theta_{3}\right|} \end{array}$$

where the oxygen pairs (O1, O4) are in opposite positions in the [TiO_6_] octahedra. (O2, O5) and (O3, O6) are positioned in the same way. The calculated Δ*d* values for [TiO_6_] octahedra with three different crystallographic positions are 0.1536, 0.2631, and 0.2909, respectively. According to the definition of P. Shiv Halasyamani, these values belong to a weak distortion range (0.05–0.40) [35]. The weak distortions of [TiO_6_] octahedra might result in a weak SHG response of the Rb_3_Ti_3_P_5_O_20_ crystal.

### 3.5. Theoretical Calculations

**Figure 7:**
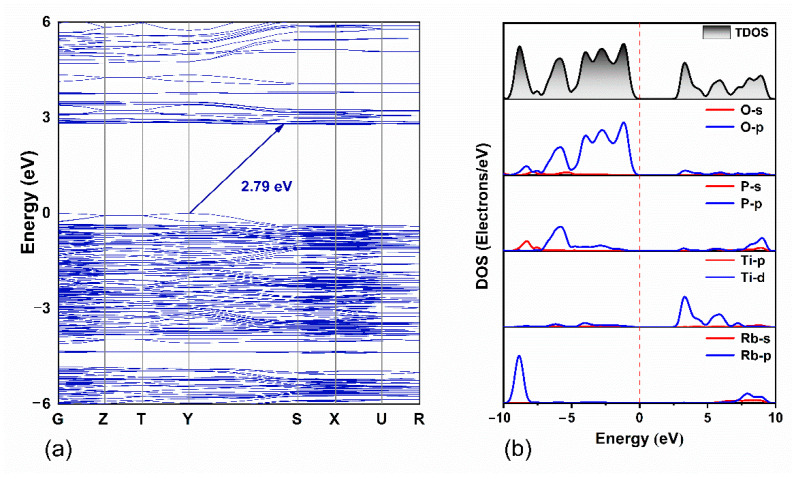
Theoretical calculation of Rb_3_Ti_3_P_5_O_20_. (**a**) Energy band structure; (**b**) TDOS and PDOS curves. The dashed line in the figure is the Fermi energy level (0.0 eV).

To explore the structure–property relationship of the Rb_3_Ti_3_P_5_O_20_ crystals, the band structure and DOS were calculated based on the DFT method. As shown in Figure 7a, Rb_3_Ti_3_P_5_O_20_ is an indirect band gap material with an energy band gap width of 2.79 eV, which is smaller than the test value (3.54 eV). It can be attributed to the underestimation of the band gap by the DFT method [36]. The calculated total densities of states and partial densities of states (TDOS and PDOS) are plotted in Figure 7b. Optical properties of crystals are closely related to electronic transitions near the Fermi level (or the forbidden band). Therefore, it is vital to investigate the valence band top (VBT) and the conduction band bottom (CBB). 

**Figure 8:**
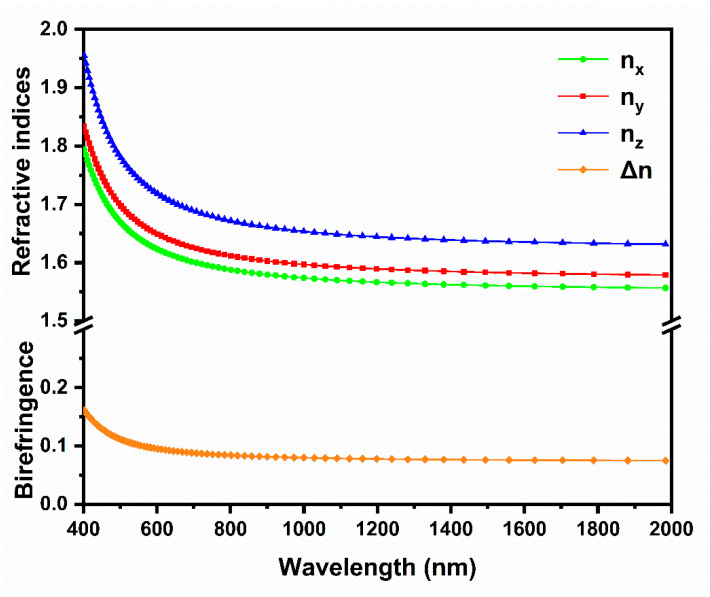
Calculated dispersive refractive index and Birefringence curves.

From the spectra shown in Figure 7b, it can be inferred that: (1) the orbital effect of alkali metal cations (Rb^+^) to electronic transitions near the forbidden band can be negligible. (2) the electronic states of VBT between –5.0 eV and 0.0 eV are mainly constituted by O 2p orbitals and a spot of P 3p orbitals. (3) the electronic states near CBB (2.7 eV–5.0 eV) are mainly contributed by Ti 3d orbitals and little component O 2p orbitals. According to these analyses, it is clear that distorted [TiO_6_] octahedra contribute considerably to the optical properties of Rb_3_Ti_3_P_5_O_20_ crystals, while [PO_4_] and [P_2_O_7_] groups contribute less to the optical properties. Based on the calculated energy band structure and DOS, linear refractive index curves and birefringence (Δn) curve of Rb_3_Ti_3_P_5_O_20_ are computed. As shown in Figure 8, the refractive indices are characterized by a considerable anisotropy and n_x_ > n_z_ > n_y_, which indicates that the Rb_3_Ti_3_P_5_O_20_ is a biaxial crystal. The values of the birefringence are 0.079 at 1064 nm and 0.104 at 532 nm. The relatively large birefringence at 1064 nm of Rb_3_Ti_3_P_5_O_20_ is close to the value of KTP (experimental Δn = 0.092 at 1064 nm).

## 4. Conclusions

In summary, large Rb_3_Ti_3_P_5_O_20_ single crystals with dimensions of 7 × 2 × 1 mm^3^ and 3 × 3 × 1 mm^3^ were successfully grown via the high temperature solution growth method in the Rb_2_O–TiO_2_–P_2_O_5_ system. The structural analysis indicates that there are two anionic groups [PO_4_] and [P_2_O_7_] in the structure of Rb_3_Ti_3_P_5_O_20_ crystal. The optical properties of the crystal, including UV-Vis diffuse reflectance spectra and transmission spectra, were studied for the first time. The results demonstrated that Rb_3_Ti_3_P_5_O_20_ displays a wide transparent range of 0.33–4.5 μm and has a relatively large band gap of 3.54 eV. The second harmonic generation measurement showed that the SHG intensity is about 0.4 × KDP. The weak distortions of [TiO_6_] octahedra might result in the weak SHG response. The band structure, the density of states, and the dispersive refractive indices were calculated by first principles calculations. The DOS spectra of Rb_3_Ti_3_P_5_O_20_ show that the valence band top is formed mainly by O 2p orbitals and the conduction band bottom is contributed to by Ti 3d orbitals. Therefore, distorted [TiO_6_] octahedra contributes considerably to the optical properties. Furthermore, Rb_3_Ti_3_P_5_O_20_ has a moderate birefringence of 0.079 at 1064 nm. This work may be used as a reference for feasible optical application prospects of Rb_3_Ti_3_P_5_O_20_ crystals.

## Figures and Tables

**Table 1:** Crystal data and structure refinement for Rb_3_Ti_3_P_5_O_20_ crystal.

Formula	Rb_3_Ti_3_P_5_O_20_
Formula mass	874.96
Temperature (K)	296.15
Crystal system	orthorhombic
Space group	Pca2_1_
a (Å)	18.2967(17)
b (Å)	6.3043(5)
c (Å)	14.7942(15)
α (°)	90
β (°)	90
γ (°)	90
Volume (Å^3^)	1706.5(3)
Z	4
Density(cal.) (g/cm^3^)	3.406
μ (mm^−1^)	10.450
F (000)	1648.0
Crystal size (mm^3^)	0.12 × 0.1 × 0.1
Radiation	Mo Kα (λ = 0.71073)
2θ range for data collection (°)	4.452 to 72.844
Index ranges	−14 ≤ h ≤ 30, −10 ≤ k ≤ 8, −24 ≤ l ≤ 24
Reflections collected	23,890
Independent reflections	8114 [R_int_ = 0.0457, R_sigma_ = 0.0540]
Data/restraints/parameters	8114/1/282
Goodness-of-fit on F^2^	1.010
Final R indexes [I ≥ 2σ (I)]	R_1_ = 0.0345, wR_2_ = 0.0826
Final R indexes [all data]	R_1_ = 0.0429, wR_2_ = 0.0864
Largest diff. peak/hole (e Å^−3^)	2.02/−1.81
Flack parameter	0.029(9)

**Table 2:** Binding energy values (eV) of Rb_3_Ti_3_P_5_O_20_, RTP, and KTP crystals.

	Rb_3_Ti_3_P_5_O_20_	RTP	KTP
Rb 3d_5/2_ (±0.1 eV)	109.3	108.9	–
Ti 2p_3/2_ (±0.1 eV)	458.6	458.6	458.4
P 2p (±0.1 eV)	132.7	132.8	132.8
O 1s (±0.1 eV)	530.1	530.4	530.9
ΔBE (O 1s−Rb 3d)	420.8	421.5	–
ΔBE (O 1s−Ti 2p)	71.5	71.8	72.5
ΔBE (O 1s−P 2p)	397.4	397.6	398.1
Reference	This work	[29]	[30]

## Data Availability

The data presented in this study are available on request from the corresponding author.
